# A Systematic Review: The Role of Artificial Intelligence in Lung Cancer Screening in Detecting Lung Nodules on Chest X-Rays

**DOI:** 10.3390/diagnostics15030246

**Published:** 2025-01-22

**Authors:** Puteri Norliza Megat Ramli, Azimatun Noor Aizuddin, Norfazilah Ahmad, Zuhanis Abdul Hamid, Khairil Idham Ismail

**Affiliations:** 1 Institut Kanser Negara, Ministry of Health, Putrajaya 62250, Malaysia; p131318@siswa.ukm.edu.my (P.N.M.R.); drzuhanis@nci.gov.my (Z.A.H.); 2 Department of Public Health Medicine, Faculty of Medicine, Universiti Kebangsaan Malaysia, Cheras 56000, Wilayah Persekutuan Kuala Lumpur, Malaysia; norfazilah@hctm.ukm.edu.my; 3 Malaysian Health Technology Assessment Section (MaHTAS), Medical Development Division, Ministry of Health, Putrajaya 62590, Malaysia; khairilidham@moh.gov.my

**Keywords:** artificial intelligence, chest X-ray, lung nodule, lung cancer, radiologist

## Abstract

**Background:** Lung cancer remains one of the leading causes of cancer-related deaths worldwide. Artificial intelligence (AI) holds significant potential roles in enhancing the detection of lung nodules through chest X-ray (CXR), enabling earlier diagnosis and improved outcomes. **Methods**: Papers were identified through a comprehensive search of the Web of Science (WOS), Scopus, and Ovid Medline databases for publications dated between 2020 and 2024. Following the Preferred Reporting Items for Systematic Reviews and Meta-Analyses (PRISMA) guidelines, 34 studies that met the inclusion criteria were selected for quality assessment and data extraction. **Results**: AI demonstrated sensitivity rates of 56.4–95.7% and specificities of 71.9–97.5%, with the area under the receiver operating characteristic (AUROC) values between 0.89 and 0.99, compared to radiologists’ mean area under the curve (AUC) of 0.81. AI performed better with larger nodules (>2 cm) and solid nodules, showing higher AUC values for calcified (0.71) compared to non-calcified nodules (0.55). Performance was lower in hilar areas (30%) and lower lung fields (43.8%). A combined AI-radiologist approach improved overall detection rates, particularly benefiting less experienced readers; however, AI showed limitations in detecting ground-glass opacities (GGOs). **Conclusions**: AI shows promise as a supplementary tool for radiologists in lung nodule detection. However, the variability in AI results across studies highlights the need for standardized assessment methods and diverse datasets for model training. Future studies should focus on developing more precise and applicable algorithms while evaluating the effectiveness and cost-efficiency of AI in lung cancer screening interventions.

## 1. Introduction

### 1.1. Background

Lung cancer remains a significant global public health issue, with substantial variation in incidence and mortality rates across different regions. While the American Cancer Society has reported a decrease in the cancer burden in the United States, China is experiencing a shift in its cancer burden epidemiology, increasingly resembling that of the United States [1]. It is estimated that approximately one-quarter of all cancers could be prevented if risk factors such as tobacco smoking and obesity were effectively controlled [2]. However, early diagnosis of the disease is crucial, as about 93% of patients are diagnosed with stage III and IV lung cancer, with an average survival time of just 18 weeks after diagnosis [3].

The National Lung Screening Trial (NLST) demonstrated that lung cancer screening has the potential to enable early detection of the disease. The study reported a 20% reduction in lung cancer mortality among participants who underwent low-dose computed tomography (LDCT) compared to those who underwent chest radiography screening [4]. However, the implementation patterns and benefits of LDCT screening vary significantly across different groups. Factors such as race, ethnicity, rurality, and socioeconomic status have emerged as major determinants of access and use [5]. Notably, the rural population undergoes fewer LDCT scans compared to the urban population [6]. In the ASEAN region, the scarcity of imaging equipment compared to global standards exacerbates these disparities [7]. Geographic accessibility is another constraint, with 5% of the target population lacking a screening facility within 40 miles, and no facilities available in rural areas at any distance [8].

Thus, new methods for interpreting chest X-rays (CXRs) using AI have been developed to address the disparities in lung cancer screening. Computer-assisted detection (CAD) developed through AI has enhanced lung cancer nodule detection on chest X-rays (CXRs) for general physicians and radiologists, leading to increased accuracy, specificity, and positive predictive value (PPV) [9]. The authors of [10] demonstrated that applying a deep learning-based automatic detection algorithm (DLAD) to CXRs improved the average area under the alternative free-response receiver operating characteristic curve for all observers from 0.67 to 0.76. The DLAD also helped detect more lung cancers (53% vs. 40%, *p* < 0.001) [10]. The performance of AI models in diagnosing CXRs has been very impressive, with median area under the curve (AUC) values ranging from 0.979 [11] to 0.983 [12] for detecting CXRs with major thoracic diseases.

While AI shows great promise in lung cancer screening, several significant challenges impact its implementation. Technical limitations include AI’s difficulty in detecting small nodules and nodules with varying densities [13]. Additionally, AI systems often underperform when applied to populations that differ from the datasets used for training [14]. Poor image quality [15] and complex anatomical structures [16] further compromise performance, particularly in detecting nodules that overlap with other structures. Moreover, AI accuracy decreases with increasing case complexity [17], and challenges arise in comparing current images with past radiographs [18]. These implementation challenges highlight the need for enhanced AI systems capable of delivering consistent performance across diverse clinical settings and patient populations.

However, it is also important to realize that AI in medical imaging was never meant to replace radiologists but rather to serve as a tool that can help them. Research revealed that AI sensitivity for detecting early-stage lung cancer can increase to 83.3% when combined with radiologists [19]. The application of AI in CXRs has the potential to help in the early identification of lung cancer and lower the mortality rate. Furthermore, AI-assisted CXR analysis could help to address some of the disparities in lung cancer screening. It could provide a less costly, potentially more available first-screening test that might be available to regions that have limited access to LDCTs or few radiologists.

### 1.2. Objective of the Review

The primary objective of this systematic literature review (SLR) is to comprehensively assess the performance of AI algorithms compared to radiologists in detecting lung nodules in CXRs. This review aims to:

1. Evaluate and compare the performance of AI algorithms against radiologists in detecting lung nodules in CXRs.
2. Analyze performance variations of AI algorithms and radiologists based on nodule sizes, morphology, and locations of the nodules.
3. Identify and examine factors contributing to discrepancies between AI and radiologist interpretations in lung nodule detection.
4. Investigate patterns of missed lung nodules when comparing AI algorithms with radiologists in chest radiograph interpretation.

## 2. Materials and Methods

### 2.1. Reference Source (PRISMA)

**Figure 1:**
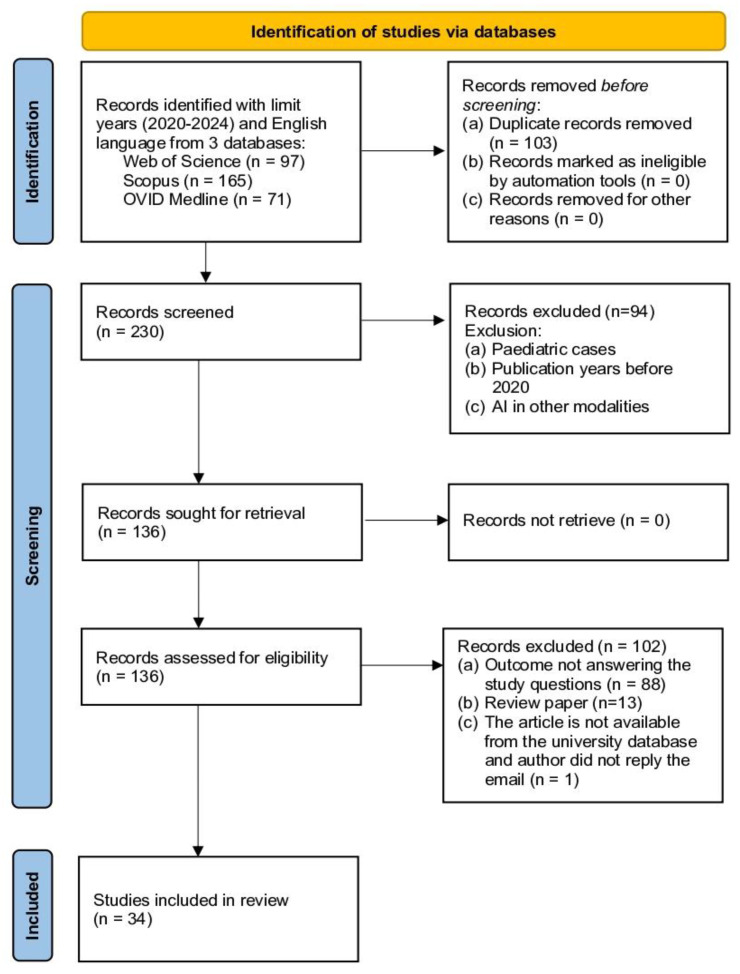
Study selection process following PRISMA guidelines.

This study adopts the Preferred Reporting Items for Systematic Reviews and Meta-Analyses (PRISMA) as the primary reference source (Figure 1). PRISMA, developed by the authors of [20], was selected due to its advantages in several aspects. Firstly, PRISMA provides researchers with clear guidelines for producing and writing SLR articles. The PRISMA checklist will guide the documentation of search strategies, study selection criteria, and data extraction processes, ensuring transparency and completeness in reporting. In addition, PRISMA supports quality control of the selected articles [20]. Referring to PRISMA has enabled researchers to develop this SLR based on four main methodological processes: namely (1) formation of research questions, (2) systematic search strategy (identification, screening, and eligibility), (3) article quality assessment, and (4) data extraction and analysis. By following PRISMA guidelines, this review aims to comprehensively present the methodological report on the current state of the study, allowing readers to evaluate the findings and their reliability for practical application.

### 2.2. Formation of Research Questions

Referring to the mnemonic PICO (Problem, Intervention, Comparison, and Outcome), the research question for this SLR was formulated. PICO is particularly suitable for medical investigations as it offers a clear and systematic means of drawing questions, which, in turn, will increase the focus and relevance of the investigation [21]. PICO improves the efficiency of literature searches, the solidity of study designs, and the dependability of evidence syntheses. In this SLR, P refers to lung cancer, I refers to the use of AI in chest radiographs, C refers to a radiologist, and O refers to lung nodule detection. This reference to the mnemonic PICO has helped the researcher to produce these research questions and the motivation behind these questions (Table 1).

### 2.3. Systematic Search Strategy

The next process involves a systematic search strategy. This process helps researchers conduct a comprehensive material search, and it has three main steps: identification, screening, and eligibility. The search utilized three electronic databases, namely Web of Science (WOS), Scopus, and Ovid Medline.

#### 2.3.1. Identification

The first author conducted the initial identification process to determine suitable keywords for this SLR. Based on the set of research questions formed, some keywords have been selected, such as “lung cancer”, “pulmonary cancer”, “lung nodule”, “pulmonary nodule”, “artificial intelligence”, “chest x-ray”, “chest radiograph”, and “radiologist”. Next, to maximize the number of articles that can be obtained, researchers have searched for synonyms of related words and variations to the keywords. These have been searched in two main sources: referring to the keywords of previous studies and keywords suggested by databases such as Ovid Medline.

This process has produced some additional keywords such as “lung neoplasm”, “pulmonary neoplasm”, “deep learning”, “machine intelligence”, “computer vision system”, “computational intelligence”, and “thoracic radiography”. In addition, researchers have also produced search strings for this SLR based on functions such as Boolean Operator, Phrase Searching, Truncation, Wild Card, and Field Code. The final search was performed on 23 July 2024. The search brought a total of 392 documents identified from the three databases (WOS Core Collection = 116, Scopus = 168, Ovid Medline = 108). The number was reduced to 333 articles using language (English) and the publication year (2020–2024) filters (WOS 97, Scopus 165, Ovid Medline 71). A total of 230 articles were selected after the duplication was removed and taken to the next process, which was screening.

#### 2.3.2. Screening

Screening is a process of filtering articles comprising several standards that the researcher uses to choose articles of interest. Two reviewers were independently involved in the screening process. Among the 230 articles that were identified and screened, 94 articles were removed based on the exclusion criteria, including pediatric cases, papers published before 2020, and articles discussing AI in other medical imaging techniques. From this screening process, there was a reduction in the articles to 136 for further determination of their relevance to the study.

#### 2.3.3. Eligibility

The eligibility process for the selection of articles is another important step of the systematic review in which the researchers take their time to assess the suitability of the selected articles about the objectives of the study. The process includes the evaluation of the title and abstract of each article and, if required, the main text. The first step is to check and ensure that the chosen articles are relevant to the research objectives and questions. In this review, the eligibility screening resulted in the removal of 102 articles for various reasons: they did not achieve the aims of the study, review articles rather than primary research, and the full text of the articles was not available in the university’s database. After the elimination process based on these criteria, 34 articles were considered to meet the inclusion criteria in the next step of the review, which was the quality assessment of the articles that were selected.

### 2.4. Evaluation of Article Quality

Next, the 34 articles selected earlier were evaluated for their quality. This process was carried out by the lead author of this SLR and assisted by two co-authors. The evaluation method by the authors of [22] was used to determine the selected articles. This method is based on six main criteria, which are as follows:

QA1. Is the purpose of the study clearly stated?

QA2. Is the interest and the usefulness of the work presented?

QA3. Is the study methodology established?

QA4. Are the concepts of the approach clearly defined?

QA5. Is the work compared and measured with other similar work?

QA6. Are the limitations of the work mentioned?

A scoring system was used to determine whether the selected articles were of good quality. Every time the researcher gave a yes answer to the evaluated criteria, 1.0 marks were given. If the researcher chooses an incomplete answer, 0.5 marks are given. If the researcher chooses the answer no, zero marks will be given. The condition for articles to be selected in this SLR is that the minimum score recorded should reach at least 3.0 and above [22]. Based on this process, the researchers found that all 34 articles that were evaluated passed the minimum score of 3.0 and were selected to be reviewed.

### 2.5. Data Extraction and Synthesis

Two reviewers independently reviewed the selected articles and extracted relevant data systematically. Most of the data collection was conducted on the results and discussion sections of the selected articles, with other sections examined where the need for context or clarity exists. The authors have utilized Petal.ai to assist with initial data extraction and manually validate all extracted data points to ensure accuracy. Data synthesis was conducted using a narrative descriptive approach since the studies included in the review are heterogeneous. The findings were organized into four main themes aligned with our research objectives. Data from all independent research were summed up in a common table for further comparison and analysis. A meta-analysis was not conducted because the study was rather diverse in terms of study designs, populations, and outcome measures. The authors used Claude.ai to improve clarity and readability under the authors’ supervision and final approval. 

## 3. Results

### 3.1. Overview of the Study

The number of research publications in AI-assisted lung nodule detection on chest radiographs, especially from 2020 to 2024, in which the number of articles has grown from 6 in 2020 to 13 in 2023 (Table 2). This SLR synthesizes the cross-national trends (Table 3) and findings from work conducted throughout this period (Table 4). These studies were conducted with participants from various geographical areas, such as Asia, including South Korea [13,17,23,24,25,26,27,28,29,30,31,32,33], Japan [16,18,34], Taiwan [35,36], and India [14,37,38]; Egypt [39], the United States [15,37,40,41], Israel [42], and Australia [43]; and European countries, such as Germany [44], France [45], The Netherlands [46,47], the United Kingdom [48,49], and Switzerland [50].

### 3.2. Study Design and Population

From the analysis of 34 selected articles, the majority employed retrospective cross-sectional study designs (*n* = 26) [15,16,17,18,24,26,28,29,30,31,32,33,34,35,36,37,38,39,41,42,43,44,45,46,48,49]. The remaining studies utilized various other methodologies, including retrospective cohort studies (*n* = 3) [25,40,47] and retrospective case-control studies (*n* = 2) [13,27].

Out of all of these study designs, only one used a randomized control trial approach [23], one conducted an experimental study using mannequin-like models or anthropomorphic phantoms [50], and one used a prospective cross-sectional study design [14]. The distribution of study designs shows that authors employed cross-sectional studies as the primary source of AI performance assessment for lung detection in chest radiographs.

Variations in sample size and the representation of study subjects were observed in the 34 studies under analysis. The largest study was conducted on 759,611 chest radiographs of 538,390 patients in five regional centers from India [38], and other select studies were conducted on 100–300 participants [24,27,35]. Some of the large sample studies sourced participants from health screenings, with 10,476 participants from a tertiary hospital [23] and 6452 participants from a healthcare screening center [25]. Control subjects were generally selected using case-control studies, like in one study with 50 subjects of lung cancer patients and 50 controls [27] and another one with 168 patients in case groups and 50 controls [13]. Certain investigations targeted higher-risk people from NLST [15] or patients with lung cancer confirmed pathologically [18].

The studies used different criteria for defining the populations they included and excluded from their research. The majority of studies involved patients who were 18 years of age and older [23,26,35,39], and a few of them were designed exclusively for patients of a certain age, namely 55–74 years, in the case of lung cancer [15]. Some of the studies confirmed nodules by CT scans or pathology [16,28,30,33], while others excluded cases according to their specific characteristics, such as extensive infiltrative lesions [31], poor image quality [39], or multiple nodules [33]. Some investigations included data collected from more than one institution or source, such as more than thirty-five centers in India [14], seven medical centers in The Netherlands [47], national institutions, and public datasets [34,36].

**Table 3:** Characteristics of selected articles.

Author and Year	Country	Study Design	Study Population	Study Setting
Nam et al., 2020 [13]	Republic of Korea	Retrospective case-control study	168 patients (mean age 71.9 ± 9.5 years) with 187 initially undetected lung cancer nodules, plus 50 normal controls; CT or X-ray validated; from March 2017 until December 2018	A single center
Sim et al., 2020 [30]	Republic of Korea	Retrospective cross-sectional study	800 radiographs (200 per center: 150 cancer cases, 50 normal); from October 2015 until September 2017	Four tertiary hospitals
Lee et al., 2020 [32]	Republic of Korea	Retrospective cross-sectional study	Two cohorts: validation (10,289 X-rays from 10,206 individuals) and screening (100,576 X-rays from 50,098 individuals)	Seoul National University Hospital
Koo et al., 2020 [33]	Republic of Korea	Retrospective cross-sectional study	378 patients (61% male; mean age 61.4 years) with ≤three pathologically proven nodules; excluding nodules < 5 mm or calcified; from January 2016 until December 2018	Tertiary hospital
Majkowska et al., 2020 [38]	India	Retrospective cross-sectional study	Two datasets: DS1 (759,611 X-rays from 538,390 patients) with natural prevalence; ChestX-ray14 (112,120 X-rays from 30,805 patients) enriched for abnormalities	Five Indian centers (DS1) and NIH database (ChestX-ray14)
Yoo et al., 2020 [15]	United States	Retrospective cross-sectional study	5485 high-risk smokers (age 55–74 years; ≥30 pack-years; 55.2% male) receiving three annual screenings; from August 2002 until April 2004	NLST multicenter trial (21 sites)
Teng et al., 2021 [35]	Taiwan	Retrospective cross-sectional study	100 subjects with CXR (47 with CT-validated nodules or masses, 53 normal); mean age 55.07 ± 13.80 years	A single center
Yoo et al., 2021 [41]	United States	Retrospective cross-sectional study	519 screening X-rays from 294 patients (98 cancer cases, 196 controls); from August 2002 until April 2004	NLST trial dataset
Homayounieh et al., 2021 [44]	Germany	Retrospective cross-sectional study	Adult patients of both genders with PA chest radiographs; including cases with nodules, non-nodular abnormalities, and normal findings	Two sites (A and B)
Tam et al., 2021 [49]	United Kingdom	Retrospective cross-sectional study	400 cases (200 tumors, 200 controls); mean ages 72.6 and 61.8 years; seven years retrospective review	NHS setting
Peters et al., 2021 [50]	Switzerland	Experiment study	61 chest phantoms with 140 solid lung nodules were placed in artificial lung parenchyma (53 phantoms with lung nodules and eight without nodules).	A phantom study using PACS-workstation
Nam et al., 2022 [25]	Republic of Korea	Retrospective cohort study	6452 health checkup participants (52% male; mean age 57.6 ± 13.0 years); a subset of 3073 with CT validation	Seoul National University Healthcare Screening Center
Bae et al., 2022 [31]	Republic of Korea	Retrospective cross-sectional study	111 patients with PA chest radiographs including 49 patients with 83 pulmonary nodules; excluding complex pathologies and CT-only visible nodules; from April 2016 until December 2019	A single center
Chiu et al., 2022 [36]	Taiwan	Retrospective cross-sectional study	Adults (≥20 years) with single lung nodule confirmed by biopsy/resection; plus, cases from four external datasets; from 2011 until 2014	Taipei Veterans General Hospital
Kaviani et al., 2022 [37]	United States	Retrospective cross-sectional study	2407 adults (≥21 years; 1248 males; mean age 39 years); de-identified CXR data; from 2015 until 2021	Eight sites (three in India, five in the US); including quaternary and community hospitals
Govindarajan et al., 2022 [14]	India	Prospective cross-sectional study	65,604 CXRs (median age 42 years); PA/AP views; excluding incomplete/artifact cases	35 centers across Six Indian states
Ahn et al., 2022 [40]	United States	Retrospective cohort study	Adult patients with specific radiograph findings (pneumonia, nodules, pneumothorax, pleural effusion)	Deaconess Medical Centre and Massachusetts General Hospital
Nam et al., 2023 [23]	Republic of Korea	Randomized control trial	10,476 adults (>18 years) undergoing chest radiography; median age 59 years; 5121 men; from June 2020 until December 2021	Tertiary hospital health screening center
You et al., 2023 [24]	Republic of Korea	Retrospective cross-sectional study	300 patients: 100 normal cases (50% male, mean age 46.5 years) and 200 with pulmonary nodules (57% male, mean age 60.0 years)	A single tertiary hospital
Hwang et al., 2023 [26]	Republic of Korea	Retrospective cross-sectional study	73 first-visit outpatients (median age 70 years) with AI-detected incidental nodules; excluding known thoracic cases	Hospital outpatient clinic
Huh et al., 2023 [27]	Republic of Korea	Retrospective case-control study	100 cases (50 pathologically confirmed lung cancers, 50 normal); December 2015 until February 2021	Seoul National University Hospital (tertiary)
Kim et al., 2023 [28]	Republic of Korea	Retrospective cross-sectional study	Training set (*n* = 998; 54% male; mean age 54.2 years) plus two validation sets (*n* = 246 and *n* = 205); including normal and nodule cases; from November 2015 until July 2019	Severance Hospital, Pusan National University Hospital and Dongsan Medical Centre
Kwak et al., 2023 [29]	Republic of Korea	Retrospective cross-sectional study	Patients with incidentally detected, pathologically proven resectable lung cancer; referred from various departments; from March 2020 until February 2022	A single center
Lee et al., 2023 [17]	Republic of Korea	Retrospective cross-sectional study	120 cases (60 biopsy-proven cancers from 5647 registry, 60 controls); expert-annotated with CT validation; from December 2015 until February 2021	Seoul National University Hospital, Korea
Ueno et al., 2023 [16]	Japan	Retrospective cross-sectional study	388 patients with suspected lung cancer; required both CT and CXR (PA view) within one-month intervals; from June 2020 until May 2022	Single institution,
Higuchi et al., 2023 [34]	Japan	Retrospective cross-sectional study	5800 CXRs total: 800 from Fukushima (50% normal, 50% nodules) and 5000 from the NIH dataset	Fukushima health centre and NIH database
Farouk et al., 2023 [39]	Egypt	Retrospective cross-sectional study	150 adults with varied lung pathologies; requiring both X-ray and CT; excluding poor-quality images and complex cases; from May 2021 until July 2022	Ain Sham University
Tang et al., 2023 [43]	Australia	Retrospective cross-sectional study	Volunteer radiologists evaluating three AI interfaces vs. no AI in paired-reader design	Royal Melbourne Hospital
Bennani et al., 2023 [45]	France	Retrospective cross-sectional study	500 patients (52% female; mean age 54 ± 19 years) with paired CXR and CT within 72 h; From emergency, inpatient, and day hospital settings; from January 2010 until December 2020	Cochin Hospital, Paris, France
Maiter et al., 2023 [48]	United Kingdom	Retrospective cross-sectional study	5722 radiographs from 5592 adults (53.8% female; median age 59 years); from July 2020 until February 2021	Sheffield Teaching Hospitals (tertiary)
Hamanaka et al., 2024 [18]	Japan	Retrospective cross-sectional study	Patients who underwent malignant lung tumor resection; from November 2021 until July 2023	Shin-Yurigaoka General Hospital
Kirshenboim et al., 2024 [42]	Israel	Retrospective cross-sectional study	683 chest radiographs from an initial pool of 50,286; excluding interpreted cases and duplicates	A single center
Topff et al., 2024 [46]	The Netherland	Retrospective cross-sectional study	25,104 radiographs from 21,039 adults (≥18 years; mean age 61.1 ± 16.2 years); Institution 1 (April 2021–February 2022) and Institution 2 (January–December 2018)	Two institutions
van Leeuwen et al., 2024 [47]	The Netherland	Retrospective cohort study	Two cohorts: 95 hand radiographs (age range 0–18 years; from January 2017–January 2022) and 140 chest radiographs (January 2012–May 2022); CT-validated reference standard	Seven Dutch hospitals

### 3.3. Performance in Lung Nodule Detection of AI Algorithms and Radiologists

Numerous studies demonstrate that AI algorithms are more sensitive than radiologists, with similar or slightly lower specificities in lung nodule detection [13,23,40]. The authors of [13] reported that AI has a significantly higher sensitivity (70% vs. 47%) and specificity (94% vs. 78%), as well as a significantly higher area under the receiver operating characteristic (AUROC) (0.899 vs. 0.634–0.663) compared to the radiologist. Similarly, another study reported superior AI sensitivity of 56.4% compared to 23.2% of radiologists, while both had relatively high specificity of 99.0% and 98.2%, respectively [23]. Additional analysis of the AI also indicated impressive AUC, where one study showed an AUC of 0.99 [32] while the authors of [37] also demonstrated a high AUC of 0.935 for missed findings. These improvements were particularly perceived to be significant in the identification of smaller nodules and those located in the ‘‘danger zone’’ [18,24].

However, a more detailed comparison of other studies shows variation in AI performance. This variability was described by [26] that depending on the specific model and data characteristics, AI sensitivities can vary from 44.1% to 95.7% and specificity from 71.9% to 97.5%. Variability in performance has also been observed for the various types of nodules, including a study that reported an AUC of 0.71 on calcified nodules but only 0.55 on non-calcified clinically important nodules [37]. This variability is further demonstrated across different studies. The authors of [28] also showed that the sensitivity of the AI model was 91.5%, in contrast to the mean sensitivity of radiologists at 77.5%, whereas the authors of [36] suggested that the sensitivity of AI was 79% with 3.04 FPs per image compared to the pooled radiologists’ sensitivity of 76%.

In radiograph classification, the sensitivity of AI was 53.4%, and specificity was 84.3%, while the performance of radiologists resulted in 38.8% sensitivity and 94.1% specificity [25]. Overall, CXR classification AUC was achieved at 0.890 by AI, 0.816 by radiologists, and a combined 0.866 [39]. However, one study reported lower values of AI sensitivity of 35% compared to the pooled radiologists’ sensitivity of 50% [50]. The use of AI systems alone could be insufficient and will be prone to over, or misdiagnosis in some of the studies [42]. Nevertheless, the most effective use of the AI system is in combination with radiologist interpretation [30,49].

The integration of AI with radiologists demonstrates an enhanced diagnostic performance. Several studies that described AI as a second reader or assistant tool show that the performance advantage was higher for junior readers [33,41,44]. The authors of [33] and [49] identified an increase in radiologist sensitivity from 78 to 91% and from 88.6 to 93.9%, respectively. Another study reported that the sensitivity ranges from 38.8 to 45.1% [25], while the authors of [44] noted that the sensitivity mean of junior radiologists increases from 40 to 52% with the help of AI. The previous studies highlighted that by implementing the AI model, the sensitivity of radiologists has risen from 77.5% to 92.1% [28]. When applying per nodule analysis, the AUC of AI was 0.875, compared to the radiologist’s AUC of 0.772 and a combined AUC of 0.834 [39].

### 3.4. AI and Radiologist Performance Across Nodule Size, Morphological Type, and Location

A study found AI improved the identification of actionable lung nodules, specifically around the missed small-sized nodules [23]. Specifically, AI was 45% accurate in identifying nodules under 1 cm in size, while human readers were only 25% accurate [13]. AI algorithms demonstrated higher accuracy with nodules of higher visibility scores, especially when nodules exceeded 2 cm [24]. However, it was found that DLAD systems improved when detecting nodules larger than 15 mm [16]. The clinical data showed that, on average, resected lung cancers were 2.6 cm in size [29].

The ability to detect lung nodules also depends on the morphological characteristics of the nodules. Several studies included nodules of various types, such as solid, part-solid, and ground-glass opacity (GGO) [16,29,33]. DLAD systems reported significantly higher sensitivity for all the solid nodules compared with part-solid and pure GGNs [16]. For calcified nodules, AI systems had higher AUC values of 0.71 than non-calcified clinically important nodules (AUC 0.55) [37]. However, both AI and radiologists have difficulty detecting GGO-predominant nodules due to their subtle radiographic features [33]. Although part-solid nodules have diagnostic challenges, a study shows that AI can detect these lesions, including invasive adenocarcinomas measuring 0.75 cm [29].

The detection performance also differentiated significantly according to the location of nodules within the chest radiograph. Studies revealed AI has higher sensitivity and specificity in detecting apical lung nodules than para-mediastinal or retro-diaphragmatic locations, which are considered “danger zones” (DZ) [24]. The authors of [13] identified that the AI system could detect lung nodules better in the apical and hilar regions than the radiologist, but both have difficulty in retro-cardiac and retro-diaphragmatic areas, with only 21% to 22% of these lesions being detected. The detection rate was overall lower in the lower lung fields at 43.8% and hilar regions at 30% [18]. In particular, smooth, well-marginated nodules without marked overlap of the internal structure were found to have significantly higher detection rates [33]. It was also found that central nodules were particularly challenging for AI-CAD systems, where sensitivity was found to be as low as 9% as compared to 64% for peripheral nodules [50].

The presence of overlapping anatomical structures provided the greatest impact on the accuracy of detection, both in the AI systems and for human readers. It was further found that 86.4% of the cases were nodules missed due to being masked by other lesions, and the majority of them were hidden by bony structures such as ribs and costochondral junctions [33]. Both AI systems and radiologists consistently struggled with nodules obscured by various anatomical structures such as bone, heart, diaphragm, and mediastinum [27]. Particularly, difficulties were observed in areas contacting the left clavicle [41], behind the heart, and hilum [49]. Anatomical overlap was most prominent in the frontal view, where nodules could be masked by anatomical normal structures [32]. To mitigate these issues, a deep learning-based synthesized bone-suppressed (DLBS) model was developed to improve nodule detection in regions where ribs and clavicles overlap without the need for special equipment or extra-radiation exposure [28]. AI also assisted less experienced individuals in detecting nodules that overlapped with anatomical structures, while readers with experience were able to detect these nodules with and without AI [41].

### 3.5. Factors Contributing to AI-Radiologist Discrepancies

Lung nodule detection presents significant challenges in both AI systems and radiologists due to nodule characteristics, anatomical structure, and technical reasons. AI systems exceed radiologists in the overall performance of detecting small nodules, but they tend to produce more false positives in their interpretations [32]. However, both AI and radiologists share common challenges. They equally struggle to identify ground-glass opacity nodules based on their subtlety and fail to examine anatomically difficult regions such as para-mediastinal, retro-cardiac, and retro-diaphragmatic areas [13,33]. Further complicating these challenges is the interpretation of chest radiographs with low contrast resolution and overlapping anatomical structures, obscuring subtle findings for both AI systems and human readers [31].

With the human factor in radiological interpretation, the performance is affected by multiple variables. The authors of [13] identified that environmental conditions such as reading time, fatigue levels, and years of experience impact radiologist performance. The impact of AI depends on both radiologist experience and case complexity [44]. Studies observe particularly high variability in performance across these experience levels and how AI assistance impacts them. For instance, AI can increase sensitivity in less experienced readers and specificity in more experienced radiologists [41].

Integration of AI into clinical practice is promising to reduce missed diagnoses but also has its challenges. A study reported that a radiologist’s accuracy can be reduced by up to 18%, and sensitivity can be reduced by 30% because of distracting findings, including COPD and small pleural effusions [49]. Different AI user interfaces (UIs) also had a major impact on how radiologists performed and interpreted AI outputs [43]. A study also described that occasional correct AI detections were rejected by readers, even where AI assistance improves outcomes [45].

The transition to practical AI in real-world clinical implementation reveals several practical limitations of current AI systems. The authors of [50] described the key challenges, including a lack of well-curated datasets, generalization, and lack of adequate publication standards for reproducibility. Another study demonstrates the lack of generalizability in training and test datasets for the AI, and the demographic differences may affect performance in real-world patient cohorts [48]. Hamanaka and Oda [18] noted the system’s main limitation, which is it cannot compare past radiographs with new ones, a key feature used in clinical practice. This research of [47] attributed the discrepancy between the results of AI and those of radiologists to the lack of clinical information and the difference between the imaging reading platform of AI and clinical practice.

### 3.6. Pattern of Missed Lung Nodule Findings: AI Versus Radiologist

Several studies have shown the ability of the designed AI algorithm to identify missed nodules. The authors of [30] confirmed the possibility of AI-improved detection of 15% of missed nodules in standalone radiologist sessions. Similarly, it was highlighted the fact that AI revealed 54% (69/131) of undiagnosed lung nodules that were missed in routine clinical practice [37]. Radiologist interpretation integrated with AI was most effective, where AI was able to identify eight out of 15 cases that all the radiologists missed. Their combination was found to have reduced the missed tumors by 60% to 65.4% [49]. Another study discovered that AI performed well in identifying right mid- and left-lower lung nodules, which are often missed by radiologists [44].

These findings were followed by other patterns in nodule detection by AI and radiologists. Comparatively, the authors of [36] showed different detection patterns. AI would have missed 68 nodules, which were detected by radiologists, while radiologists failed to detect 91 nodules detected by AI. The authors also highlighted that none of the AI or radiologists was able to identify nodules in 89 patients during early assessment [36]. The authors of [50] further described that while the adopted AI-CAD systems achieved detection of the lesions that the radiologists missed, the overall sensitivity was poorer than that of radiologists. Meanwhile, a study observed comparable disparities in obscured regions where AI and radiologists exhibited similar miss rates, with lung nodules missed by AI in 38.7% and by radiologists in 38% [39].

Nevertheless, specific challenges were reported in AI missed cases. Several cases of AI failures with GGO-predominant nodules were reported [33]. For instance, in the case of a 75-year-old man, AI failed to detect the tumors, as well as two radiologists. Further, AI occasionally missed subsolid nodules with spiculated edges in the left upper lobe [33]. The authors of [48] found that AI failed to detect findings in real-world patient cohorts more than radiologists for lung nodule cases. Lung cancer detection specifically showed varying miss rates. The AI missed 12/48 lung cancer cases, whereas radiologists failed to identify 7/48 cases, and four cases were missed by both AI and radiologists [15].

Another study stated that AI failed to identify all 18 cases of adenocarcinoma in situ (AIS) and three additional cases because of the flat appearance of tumors in radiographic interpretation [18]. Concurrently, AI-reported missed findings in 21.1% (5289/25,104) of cases, of which 0.9% (47/5289) were clinically significant; among the missed findings were 71.4% (25/35) of lung nodules [46]. It was found that AI had a detection rate of around 1% for unexpected nodules in initial CXRs, 70% of which were true positive nodules, and 20.5% needed further action [26].

**Table 4:** AI versus radiologist performance in lung nodule detection across study parameters and outcomes.

Author and Year	Performance			Nodule Characteristics (Morphology Type, Size, and Location)		Discrepancies Factors Between AI and Radiologists in Lung Nodule Detection	The Pattern of Missed Lung Nodule Finding
	AI	Radiologist	Combined	AI	Radiologist		
Nam et al., 2020 [13]	Sensitivity: 70% Specificity: 94% False positive rate: 21.1% (46 of 218) AUROC: 0.899 Software: Lunit INSIGHT CXR version 1.0.1.1 (Lunit Inc., Seoul, Republic of Korea)	Sensitivity: 47% Specificity: 78% False positive rate: 19.0% (166 of 872) AUROC: 0.634–0.663	AI-aided radiologists improve AUROC to 0.685–0.724	Detection rate of nodule conspicuity category 3: 93% Consistent performance across different size nodules (AI detects 45% of <1.0 cm nodules compared to radiologists with a 25% detection rate) Better detection at apical and hilar region compared to radiologist Struggle with retro-cardiac and retro-diaphragmatic area	Detection rate of nodule conspicuity category 3: 70% Improved detection with larger nodules Struggle with apical, hilar, retro-cardiac, and retro-diaphragmatic area	AI challenges: Small nodules (Category 0) Density of nodule Radiologist challenges: Certain location An environmental condition during reading Level of fatigue Years of experience	N/A
Sim et al., 2020 [30]	Sensitivity: 67.3% FPPI: 0.2 Software: Samsung Auto Lung Nodule Detection-ALND, version 1.00 (Suwon, Republic of Korea)	Sensitivity: 65.1% FPPI: 0.2	Radiologists’ sensitivity with DCNN: 70.3% FPPI: 0.18	N/A	The lower detection rate in smaller nodules (1–2 cm) compares to larger nodules (2–3 cm) with/without DCNN. The greater nodule detection rate in the peripheral lung compared to in the lung projecting over the mediastinum or diaphragm with or without DCNN	The DCNN software improved the sensitivity of radiologists, irrespective of radiologist experience, nodule characteristics, or the vendor of the radiography acquisition system	AI helped find 15% of missed nodules from standalone sessions
Lee et al., 2020 [32]	Validation test performance: Sensitivity: 90% Specificity: 97% NPV: 100% PPV: 2.7% AUC: 0.99 False-positive rate: 3.1% Screening cohort performance: Cancer-Positive Radiographs: Sensitivity: 40% (39/98) Specificity: 97% NPV: 100% PPV: 1.3% AUC: 0.78 2. Visible Lung Cancers: Sensitivity: 83% (39/47) Specificity: 97% NPV: 100% PPV: 1.3% AUC: 0.97 3. Visible Lung Cancers: Sensitivity: 100% (28/28) Specificity: 97% NPV: 100% PPV: 0.9% AUC: 0.99 (95% CI: 0.99–0.99) Software: Lunit INSIGHT CXR version 4.7.2 (Lunit Inc., Seoul, Republic of Korea)	Sensitivity: 60% Specificity: 100% NPV: 100% PPV:19% False-positive rate: 0.3%	N/A	N/A	N/A	AI performance improved with increased visibility of lung cancer in radiographs	N/A
Koo et al., 2020 [33]	Sensitivity: 67.3% Specificity: 86.2% False positives per image: 0.2 AUROC for abnormality detection: 0.87–0.96 Software: Lunit INSIGHT CXR, version 1.00 (Lunit Inc., Seoul, Republic of Korea)	Average radiologist sensitivity without AI: 88.6% Expert radiologists’ sensitivity: 40% to 87% False positives per image: 0.063 AUROC for Group 1 Radiologist: 0.93 AUROC for Group 2 Radiologist: 0.96	Average radiologist sensitivity with AI: 93.9% (5.2% increase) False positives per image with AI: 0.032 The number of false positives per image fell significantly when radiologists were assisted by the DCNN	DCNN is better at the detection of nodules larger than 5 mm, solid, round-shaped, well-marginated, and laterally located nodules. DCNN struggles with masked nodules due to overlapping structure and GGO-dominant nodules	The radiologist missed a masked nodule due to overlapping with bony lesions and a GGO-dominant nodule in the upper left lobe	Radiologist interpretation errors due to: Poor spatial resolution Lesional masking by anatomical structures Inadequate radiological skills Overwork DCNN limitations: Unreliable differentiation between true and false positives Performance affected by nodule size and nature	AI missed a GGO-dominant nodule in a 75-year-old man (also missed by two radiologists) AI missed subsolid nodule due to GGO predominance One radiologist failed to re-detect a nodule when AI data were available AI missed a subsolid nodule with a spiculated margin in the left upper lobe Missed spiculated nodule, diagnosed as adenocarcinoma
Majkowska et al., 2020 [38]	Sensitivity CXR14 dataset: 82.4% DS1 dataset: 44.1% Specificity CXR14 dataset: 84.9% DS1 dataset: 97.5% PPV CXR14 dataset: 49.2% DS1 dataset: 77.7% AUC CXR14 dataset: 0.91 DS1 dataset: 0.72 CNN architecture: Xception	Sensitivity CXR14 dataset: 79.7% DS1 dataset: 40.1% Specificity CXR14 dataset: 82.3% DS1 dataset: 96.7% PPV CXR14 dataset: 44.3% DS1 dataset: 72.4% AUC: N/A	N/A	N/A	N/A	Lack of ground truth labels leads to inconsistent performance across different datasets Risk of mislabeling or missing critical findings of difficult cases due to single-reader approach limitations and majority-vote approach	N/A
Yoo et al., 2020 [15]	All radiograph Sensitivity: 86.2% Specificity: 85% Digital radiograph Sensitivity: 96.0% Specificity: 93.2% Malignant nodule detection: Sensitivity: 100% Specificity: 90.9% NPV: 100% PPV: 8.2% Software: Lunit INSIGHT CXR, version not stated (Lunit Inc., Seoul, Republic of Korea)	All radiograph Sensitivity: 87.7% Specificity: 86.7% Digital radiograph Sensitivity: 88.0% Specificity: 82.8% Malignant nodule detection: Sensitivity: 94.1% Specificity: 91% NPV: 99.9% PPV: 7.8%	N/A	N/A	N/A	The inferior quality of chest radiographs led to the underperformance of AI AI struggled with non-calcified nodule detection AI was trained on data obtained after 2010, while NLST data were from 2002 to 2004 Ground truth labels created without CT correlation NLST radiologist performance based on pooled data (may not reflect real-world variation)	Missed cases in nodule detection AI 9/65 cases Radiologist 8/65 cases Both: 1 case Missed cases in cancer detection AI 12/48 cases Radiologist 7/48 cases Both 4 cases Missed cases in malignant nodules AI 2/34 cases Radiologist 2/34 cases Both 0 cases
Teng et al., 2021 [35]	Sensitivity of mass probability algorithm: 81% Specificity of mass probability algorithm: 90.57% AI mass probability algorithm AUC: 0.916 (95% CI 0.891–0.937) Sensitivity of heat map algorithm: 38.30% Specificity of heat map algorithm: 98.11% Heat map algorithm AUC: 0.778 (95% CI 0.743–0.811) Software: QUIBIM Chest X-ray Classifier (Valencia, Spain)	Sensitivity of trained radiographers: 77.30% Specificity of trained radiographers: 78.30%	N/A	N/A	N/A	N/A	N/A
Yoo et al., 2021 [41]	N/A	Radiology residents Sensitivity: 61% Specificity: 88% FPPI: 0.15 Radiologist Sensitivity: 76% Specificity: 79% FPPI: 0.24	Radiology residents with AI Sensitivity: 72% Specificity: 88% FPPI: 0.12 Radiologist with AI Sensitivity: 76% Specificity: 86% FPPI: 0.17 Software: Lunit INSIGHT CXR version 2.4.11.0 (Lunit Inc., Seoul, Republic of Korea)	AI identified subtle lesions missed on prior radiographs which is useful for low visibility and overlapping lesions Has lower performance on conventional radiographs	Improved with AI to detect subtle lesions, especially in less-experienced reader	AI benefits for different experience levels: Improved sensitivity of less experienced reader Improve the specificity of more experienced readers	Notably, 7 out of 98 cancer-positive patients had missed lung cancer on prior CXRs AI detected more missed lung cancers than resident radiologists (71% vs. 39%, *p* = 0.021) No significant improvement for experienced radiologists (51% vs. 57%, *p* = 0.63) AI still missed some lung nodules on CXRs
Homayounieh et al., 2021 [44]	N/A	Junior radiologist mean sensitivity: 40% Senior radiologist mean sensitivity: 51% Junior radiologist mean specificity: 95% Senior radiologist mean specificity: 90% Junior radiologist means partial AUC: 0.73 Senior radiologist means partial AUC: 0.71	Junior radiologist mean sensitivity: 52% Senior radiologist mean sensitivity: 60% Junior radiologist mean specificity: 96% Senior radiologist mean specificity: 94% Junior radiologist means partial AUC: 0.78 Senior radiologist means partial AUC: 0.76 Software: AI-Rad Companion Chest X-ray Algorithm (Siemens Healthineers AG, Erlangen, Germany)	AI improved detection across all difficulty nodule levels (easy, moderate, and challenging)	N/A	The impact of AI assistance is not constant depending on the radiologist’s experience and the complexity of the cases Challenging nodules likely difficult for both AI and humans	AI detected right mid- and left-lower lung nodules missed by radiologists
Tam et al., 2021 [49]	Sensitivity: 80% Specificity: 93% Accuracy: 87% Software: Red Dot (Behold.ai, London, UK)	Mean sensitivity: 78% Mean specificity: 96% Mean accuracy: 87%	Mean sensitivity: 91% Mean specificity: 89% Mean accuracy: 90%	Overall sensitivity increases with tumor size 1–2 cm	N/A	Radiologist has reduced accuracy by 18% and sensitivity by 30% due to distracting findings (e.g., COPD, small pleural effusion) Another factor for missed cases includes small tumor size, poor film quality, and hidden tumor	AI detected 8 out of 15 cases missed by all radiologists AI detected 70.2% of tumors missed by at least one radiologist Combined AI and radiologists reduced missed tumors by 60–65.4%
Peters et al., 2021 [50]	Sensitivity: 35% Specificity: 84% Software: InferRead®DR (Infervision Technology Ltd., Beijing, China)	Pooled sensitivity: 50% Pooled specificity: 86%	N/A	AI-CAD better performing on peripheral nodules (sensitivity of peripheral vs central: 0.64 vs. 0.094) (*p* = 0.025)	Radiologist sensitivity by nodule size: - 10–12 mm: 0.80 - 8 mm: 0.66 (*p* = 0.04) - 5 mm: 0.14 (*p* < 0.001) Radiologist sensitivity by nodule location (peripheral vs central): 0.66 vs. 0.48 (*p* = 0.004)	AI-CAD systems use chest phantoms instead of human patients, which leads to difficulties with ancillary findings Factors contributing to discrepancies include the need for good datasets, limitations in generalization, and insufficient publication standards for reproducibility	AI-CAD identified lesions missed by radiologists although had reduced overall sensitivity compared to radiologists
Nam et al., 2022 [25]	Sensitivity: 53.4% Specificity 84.3% AUROC: 0.748 Detection rate: 37.7% False positive rate 0.36/radiograph Software: Lunit INSIGHT CXR version 2.0, Lunit Inc, Seoul, Republic of Korea	Sensitivity: 38.8% Specificity: 94.1% Pooled AUROC: 0.671 Detection rate 32% False positive rate 0.09/radiograph	Radiologists’ sensitivity to AI: 45.1% Radiologists’ specificity with AI: 92.2% The detection rate with AI is 38.9% Only a minimal increase of 0.24% detection rate when radiologists aided with AI	The median nodule detection size was 1.2 cm	N/A	N/A	N/A
Bae et al., 2022 [31]	N/A	Sensitivity use of CXR alone: Reader 1 (R1): 60.2% (50/83) Reader 2 (R2): 53% (44/83)	Sensitivity withBSt-DE: Reader 1: 89.2% (74/83) Reader 2: 83.1% (69/83) Sensitivity with BSp-DL: Reader 1: 78.3% (65/83) Reader 2: 71.1% (59/83)	BSt-DE has good performance with small nodules, bone overlapping, and peripheral location	N/A	Challenges in 2D image interpretation due to low contrast resolution and overlapping anatomical structures AI training data limitations Radiologist experience and interpretation variability	
Chiu et al., 2022 [36]	AI model sensitivity: 79% with false positive per image 3.04 The AI model detected nodules 19 (0–199) days early Deep Learning architecture: You Only Look Once version 4 (YOLOv4)	Expert screening sensitivity is approximately 76% The radiologists detected nodules only eight (0–263) days early	N/A	AI has low sensitivity (50%) with smaller nodules < 1 cm. Improve detection with larger nodules	N/A	The AI model has potential overestimation due to the influence of the training data (data size and quality) The study used written reports by the radiologists, which might underestimate the doctor’s performance	AI-detected lung nodules early in 91 cases missed by radiologists Radiologists detected 68 cases of nodules missed by AI 89 patients’ nodules were not detected early by either AI or radiologists
Kaviani et al., 2022 [37]	AI AUC for missed findings up to 0.935 AI AUC for calcified nodules: 0.71 AI AUC for non-calcified, clinically important nodules: 0.55 AI identified >99% of nodules with 0.2 false positives per image Software: qXR (Qure.Ai Technology, Mumbai, India)	N/A	N/A	AI has higher performance on detection of calcified nodules—calcified nodules (AUC 0.71) vs. non-calcified nodules (AUC 0.55)	N/A	Radiologist challenges: Often subtle or difficult to detect Some detected by AI, not in original reports AI challenges: Lower AUCs for some missed findings due to the increased complexity of subtle findings	AI detected 69/131 (53%) of missed lung nodules
Govindarajan et al., 2022 [14]	AI performance in categorizing normal and abnormal CXR (use radiologist as reference standard) Sensitivity: 87.9% Specificity: 82.9% AUROC: 87.1% NPV: 98.9% AI performance in categorizing CXR into sub abnormality (in this case lung nodule) Sensitivity: 71.9% Specificity: 95.5% AUROC: 91.5% NPV: 99.8% Software: qXR (Qure.Ai Technology, Mumbai, India)	N/A	Turnaround time of radiologists is reduced with AI assistance specifically: Without AI (pre-qXR): 83.028 min average TAT With AI (post-qXR): 50.287 min average TAT Overall reduction: 32.741 min (40.63% decrease)	N/A	N/A	AI has poor generalization due to target domain divergence leading to AI-radiologist discrepancies	N/A
Ahn et al., 2022 [40]	Sensitivity: 81.6% Specificity: 73.1% AUROC: 0.858 Software: Lunit INSIGHT CXR, version 3.1.2.0 (Lunit Inc., Seoul, Republic of Korea)	Sensitivity: 56.7% Specificity: 88.5% AUROC: 0.724	Reading time improved with AI assistance with an overall reduction was 3.9 s (*p* < 0.001)	N/A	N/A	Factors that influence reader performance with AI: Size of finding Extent of findings Number of findings Impact of extensive AI output: May slow down readers Could contribute to interpretation discrepancies	N/A
Nam et al., 2023 [23]	Sensitivity for actionable lung nodules: 56.4% Specificity for actionable lung nodules: 99.0% False-referral rate: 45.9% Positive-report rate: 2.3% Software: Lunit INSIGHT CXR version 2.0.2.0 (Lunit Inc., Seoul, Republic of Korea)	Sensitivity for actionable lung nodules: 23.2% Specificity for actionable lung nodules: 98.2% False-referral rate: 56% Positive-report rate: 1.9%	AI-based CAD improved actionable lung nodule detection (odds ratio: 2.4)	AI improved the detection rate of Solid nodules that are larger than 8 mm Subsolid nodules where the solid component is larger than 6 mm Small-sized nodules that might be overlooked during normal review Malignant lung nodules with a higher detection rate compared to those without AI	N/A	N/A	N/A
You et al., 2023 [24]	Sensitivity of danger zone (DZ): 64.2% Sensitivity of non-danger zone (NDZ): 83.2% Software: Med-Chest X-ray System, version 1.0.1, (VUNO, Seoul, Republic of Korea)	N/A	Non-expert radiologists: improved DZ nodule detection with DLD	DLD has better performance for: Apical lung nodules vs. para-mediastinal/retro-diaphragmatic Higher nodule visibility scores Nodules > 2 cm DLD has poorer performance in DZ nodules vs. NDZ (despite DZ nodules being larger)	N/A	Factors affecting DLD performance: Trained based on human-provided chest radiographs Potential human bias in training data Data quantity vs. performance: Large training dataset (15,809 CXRs) Still showed poorer DZ nodule detection	N/A
Hwang et al., 2023 [26]	Sensitivity: 44.1–95.7% Specificity: 71.9–97.5% Software: Lunit INSIGHT CXR version 3, Lunit Inc., Seoul, Republic of Korea)	N/A	N/A	False positive due to: Bone overlaps/bony lesions Vascular structure Lymph nodes Atelectasis Fluid collection (pulmonary edema/pulmonary effusion)	N/A	Imaging challenges: Different pathologies may present similar findings Difficulty in differentiation	The AI-detected unexpected nodules in ~1% of initial CXRs with 70% of true positive nodules and 20.5% needing management
Huh et al., 2023 [27]	N/A	Expert performance (seven experts) Sensitivity: 67.3% Specificity: 100%	Radiologist with DLAD sensitivity: 72.8% Radiologist with DLAD specificity: 94.2–97.7 Software: Lunit INSIGHT CXR version 4.7.2 (Lunit Inc., Seoul, Republic of Korea)	Limitations in the detection of those non-visible malignant nodules or obscured by anatomical structure (bone, heart, diaphragm, and mediastinum)	Limitations in the detection of those non-visible malignant nodules or obscured by anatomical structure (bone, heart, diaphragm, and mediastinum)	The human interpretation introduces intra/inter-observer variability, leading to AI evaluation biases The characteristic of expert-determined vs gold standard affects the accuracy of AI performance evaluation Characteristics of experts and radiologists such asDetection of only clearly visible lung nodules can lead to interpretation discrepancies	N/A
Kim et al., 2023 [28]	DLBS model sensitivity: 91.5% (109/119) Original model sensitivity: 79.8% (95/119)	Radiologists’ mean sensitivity: 77.5%	Radiologists with DLBS model sensitivity: 92.1% Radiologist performance with DLBS assistance can reduce false-positive markings per image	DLBS model improved detection across nodule types by suppressing bone structures (rib, clavicle, and other anatomic structures)	N/A	N/A	N/A
Kwak et al., 2023 [29]	AI detection rate: 100% (13/13) Stage I detection: 7 patients (53.8%)—requiring only surgery Median abnormality score: 78% (range: 26–95%) Software: Lunit INSIGHT CXR, version 2 and 3 (Lunit Inc., Seoul, Republic of Korea)	N/A	N/A	Nodule Characteristics detected by AI: Median size of resected lung cancer: 2.6 cm Notable detection of small invasive adenocarcinoma (0.75 cm) Detect both solid and part-solid nodules	N/A	N/A	N/A
Lee et al., 2023 [17]	High Diagnostic Accuracy (full dataset) AI Performance: Per-radiograph sensitivity: 80% Per-lesion sensitivity: 73% Specificity: 97% AUC: 0.88 AUFROC: 0.85 Low Diagnostic Accuracy (10% dataset) AI Performance: Per-radiograph sensitivity: 58% Per-lesion sensitivity: 47% Specificity: 95% AUC: 0.77 AUFROC: 0.71 CNN architecture: ResNet34	Per-lesion sensitivity: 53% Specificity: 88% AUC: 0.77 AUFROC: 0.71	Readers with high diagnostic accuracy AI: Per-lesion sensitivity: 82% Specificity: 94% AUC: 0.82 AUFROC: 0.79 Readers with low diagnostic accuracy AI (no significant improvement): Per-lesion sensitivity: 50% Specificity: 93% AUC: 0.75 AUFRC: 0.72	N/A	N/A	The performance of both AIs reduced significantly as diagnostic difficulty increased However, high diagnostic accuracy AI maintained better performance across all difficulty	N/A
Ueno et al., 2023 [16]	Sensitivity of AI: 68.9% Sensitivity of AI: 68.9% False positive rate of AI: 0.168 Software: EIRL Chest X-ray Lung Nodule (LPIXEL Inc., Tokyo, Japan)	N/A	N/A	DLAD has a lower detection rate for small-sized nodules and part solid or pure ground-glass nodules (GGNs) DLAD has a higher detection rate for solid nodules and nodules larger than 15 mm Reduce detection for medial side of right lower lung, lateral side of right upper lung, and left lower lung area overlapping with heart Additional factors affecting detection include nodules with a visibility score of two (not well visible)	N/A	Challenges for radiologists: Manual detection difficulties Varying nodule visibility Presence of overlapping structures DLAD detection difficulties: Small-sized nodules Subsolid nodules Nodules with overlapping structures	N/A
Higuchi et al., 2023 [34]	Sensitivity Type A dataset: 75% Type B dataset: 72% Specificity Type A dataset: 60% Type B dataset: 74% AUC Type A dataset: 0.74 Type B dataset: 0.79 CNN architecture: CheXNet model	Sensitivity: 47.1% Specificity: 96.4% AUC: 0.72	N/A	N/A	N/A	A massive teaching dataset is needed to increase the accuracy of the AI	N/A
Farouk et al., 2023 [39]	Detection rate: 61.3% AUC per nodule analysis: 0.875 AUC per CXR classification: 0.890 Software: Lunit INSIGHT CXR version 2.4.11.0 (Lunit Inc., Seoul, Republic of Korea)	Detection rate: 61.3% AUC per nodule analysis: 0.772 AUC per CXR classification: 0.816	Detection rate: 62% AUC per nodule analysis: 0.834 AUC per CXR classification: 0.866	Detection rate for right middle lung zone: 21.3% Detection rate for left lower lung zone: 11.2% High sensitivity of solid nodule: 91.4% (superior to visual and combined visual/AI) Poor performance on GGNs compares to combined visual or AI	Detection rate for right middle lung zone: 22.4% (22.1% if combined with AI) Detection rate for left lower lung zone: 12.9% (11.6% if combined with AI)	AI and radiologists cannot detect nodules at blind or obscured areas on CXR	AI missed lung nodules in 38.7% of cases Radiologists missed lung nodules in 38% of cases
Tang et al., 2023 [43]	N/A	Sensitivity of radiologists without AI: 64% Specificity of radiologists without AI: 88% AUC of radiologist without AI: 0.82	Sensitivity of radiologist with UI-A (text-only): 67% UI-B (text + AI confidence score): 60% UI-C (text + AI confidence score + image overlay): 63% Specificity of radiologist with UI-A (text-only): 85% UI-B (text + AI confidence score): 81% UI-C (text + AI confidence score + image overlay): 90% AUC of radiologist with UI-A (text-only): 0.87 (significant improvement, *p* < 0.001) UI-B (text + AI confidence score): 0.77 (slight decrease, not significant) UI-C (text + AI confidence score + image overlay): 0.80 (slight decrease, not significant) Software: Annalise Enterprise CXR (Annalise.ai, Sydney, Australia)	N/A	N/A	Different UIs affect radiologist performance Radiologist performance varied with different AI output UIs	N/A
Bennani et al., 2023 [45]	N/A	Sensitivity without AI Thoracic radiologist 51.8% General Radiologist 38.1% Radiologist residents 36.1% All readers 42.0% Specificity without AI Thoracic radiologists 89.2% General Radiologist 90.8% Radiologist residents 88.3% All readers 89.4%	Sensitivity with AI Thoracic radiologists 55.9% General Radiologist 55.7% Radiologist residents 49.7% All readers 53.8% Specificity with AI Thoracic radiologists 90.6% General Radiologist 91.8% Radiologist residents 92.1% All readers 91.5% Software: ChestView version 1.2.0 (Gleamer, Paris, France)	AI increase detection of small nodules that are usually overlooked	N/A	Reader-AI interaction: Some correct AI detections were rejected by readers Possible mistrust of AI suggestions Reading time: 25 s decrease with AI assistance	AI missed the left lower lobe nodule (confirmed by CT) Only one thoracic radiologist detected the missed nodule AI reported a false-negative for the left lower lobe
Maiter et al., 2023 [48]	Compared with suspicious nodules: Sensitivity 54.5% Specificity: 83.2% PPV: 5.5% NPV: 99% Accuracy: 82.7% FPPI: 0.18 Compared with cancer diagnosis: Sensitivity 60.9% Specificity: 83.3% PPV: 5.6% NPV: 99.2% Accuracy: 82.9% FPPI: 0.18 Software: ALND version 1.0 (Samsung Electronics, Suwon, Republic of Korea)	Clinical reports sensitivity: 80.0% Clinical reports specificity: 98% Clinical reports PPV: 35.7% Clinical reports NPV: 99.7% Clinical reports accuracy: 97.5% FPPI: 0.02	N/A	N/A	N/A	AI challenges: Underperformance in real-world patient cohort Lack of generalizability in training/testing datasets Low PPV may lead to over-investigation and limit clinical practice translation Demographic differences may affect performance	AI missed more findings than radiologists in lung nodule detection
Hamanaka et al., 2024 [18]	AI detection rate: 51.7% of all patients (90/174) 57.7% excluding adenocarcinoma in situ cases Software: EIRL X-ray Lung Nodule version 1.12 (LPIXEL Inc., Tokyo, Japan)	N/A	N/A	AI sensitivity by lung area: - Lung apices: 60% - Upper lung field: 63.6% - Middle lung field: 78.6% - Lower lung field: 43.8% - Hilar area: 30% AI sensitivity by tumor diameter: - ≤10 mm: 0 - 11–15 mm: 0.38 - 16–20 mm: 0.52 - ≥21 mm: >83% Lower lung fields were less frequently detected	N/A	AI challenges include overlapping anatomical structures, small nodules (<0.7 cm), oversight, and specific tumors such as adenocarcinoma in situ (AIS). Larger total histopathological tumor size improves AI detection Physician challenges include detecting smaller tumors (<1 cm) and GGNs AI is unable to make comparative diagnoses with past radiographs	AI missed all 18 cases of AIS AI missed 3 cases due to flat-appearing tumors on radiographs
Kirshenboim et al., 2024 [42]	Sensitivity: 92% Specificity: 95% Software: Lunit INSIGHT CXR version 2.0.2.0 (Lunit Inc., Seoul, Republic of Korea)	N/A	AI improves radiologist performance, especially for less skilled interpreters Experienced chest radiologists have similar performance with/without AI	N/A	N/A	AI software limitations: The false interpretation of nodules is due to subtle findings such as nipple shadows, skeletal abnormality, foreign objects, or other non-pulmonary findings May be insufficient without a formal radiology report Potential for over-diagnosis or misdiagnosis	Clinician commented on 386 CXRs (56.5%). True nodule detection by clinician were 113 CXRs (16.5%) 31 CXRs (4.5%)—incorrectly identified false nodules as real nodules 242 CXRs (35%)—clinician no mention of AI-detected nodules Of these, 68 patients (10%) needed follow-up workup
Topff et al., 2024 [46]	N/A	N/A	N/A	The AI software (ChestEye Quality, Oxipit, Vilnius, Lithuania) identified nodular opacities, the most frequent unreported findings, and solitary lung lesions	N/A	AI software flagged reports with less descriptive language AI limitations include no access to the patient’s prior imaging, which could hinder the ability to detect missed findings in follow-up examinations	AI detected discrepancies between imaging and report in 21.1% of cases (5289/25,104) Clinically relevant missed findings were 0.9% of cases (47/5289) with lung nodules 71.4% (25/35) Institution-confirmed clinically relevant missed findings were 74.5% (35/47)
van Leeuwen et al., 2024 [47]	Sensitivity: 64% to 89% Specificity: 80% to 99% AUC ranged: 0.86 to 0.93 *** Software used	Sensitivity: 89% Specificity: 80% Mean AUC: 0.81	N/A	Decrease performance with lower nodule conspicuity Nodule size had a limited correlation with performance for most algorithms	Decrease performance with lower nodule conspicuity	The difference in image reading platforms compare to clinical practice and absence of clinical information may contribute to discrepancies between AI and radiologists	N/A

Notes: Abbreviation: N/A, Not available; AI, Artificial Intelligence; AUC, Area under the curve; AUROC, Area under the receiver operating characteristic; BSp-DL: Bone suppression deep learning, BSt-DE, Bone subtraction dual energy; CAD, Computer-assisted detection; CI, confidence interval; COPD, Chronic obstructive pulmonary disease; CNN, Convolutional Neural Network; CT, Computed tomography; CXR, Chest X-ray; DCNN, Deep convolutional neural network; DLAD, Deep learning-based automatic detection; DLD, Deep learning detection; DZ, Danger zone; EMR, Electronic medical record; FPPI, False positive per image; FRCR, Fellow of Royal College of Radiologist; GGO, Ground-glass opacity; MDT, Multidisciplinary team; NDZ, Non-danger zone; NPV, Negative predictive value; NLST, National Lung Screening Trial; PACS, Picture archiving and communication system; PET-CT, Positron emission tomography-computed tomography; PPV, Positive predictive value; UI, User interface. *** Software used in this study are as follows: Annalise Enterprise CXR v3.1, Annalise.ai, Sydney, Australia; InferRead DR Chest v1.0.0.1, Infervision, Beijing, China; Lunit INSIGHT CXR v3.1.4.4, Lunit Inc., Seoul, Republic of Korea; Milvue Suite-Smart Urgences v1.24, Milvue, Paris, France; ChestEye v2.6, Oxipit, Vilnius, Lithuania; AI-Rad Companion Chest X-ray v9, Siemens Healthineers, Erlangen, Germany; Med-Chest X-ray v1.1.x, VUNO, Seoul, Republic of Korea.

## 4. Discussion

The integration of AI in chest radiographs for lung cancer screening plays an important role due to the high global burden of lung cancer and the disparities of traditional screening approaches using LDCT. The evidence presented shows that AI algorithms generally obtain higher sensitivity (ranging from 56.4% to 95.7%) than radiologists (ranging from 23.2% to 76%) while maintaining comparable specificity [13,23,25,26,29,36]. This performance characteristic is highly important in screening settings, where early detection is critical. However, the 44.1% to 95.7% variability in the sensitivity of the AI model [26] is concerning and asks pertinent questions about standardization and reliability. The amount of variability in the AI performance is strongly related to the specific model, implementation context, and characteristics of the data used for training. Therefore, this highlights the need for standardized protocols to assess the AI performance and training datasets that accurately reflect the population to be screened.

The differential performance of AI systems across nodule characteristics has big implications for screening programs. AI performs better than radiologists for larger nodules (i.e., >2 cm) and solid nodules [13,16,24,37] but has considerable variation in performance for different nodule morphology and location. Current AI implementations may miss subtle early-stage cancers that are critical targets in screening programs based on the lower detection rates for GGO nodules [16,33] and nodules in anatomically challenging locations (such as the “danger zone”) [24]. Since these subtle findings are mostly early-stage lung cancer with a better prognosis if diagnosed early, this limitation is particularly concerning. Future AI development should, therefore, address these performance gaps to maximize the efficacy of such screening programs in capturing the full range of actionable findings.

The screening context offers both opportunities and challenges in the complementary relationship of AI and radiologists. Using AI as a second reader increased sensitivity by 5.3% to 15% [25,28,33,44,49] and may benefit the screening programs, especially for less experienced readers. Yet, the variable performance across different clinical contexts [26] and levels of experience [13,41,44] implies that thoroughly evaluating the integration of AI into current screening workflows is necessary. While there is potential for AI to improve missed diagnoses, it must be balanced with a risk of over-investigation, particularly given the reported PPV of 5.1% [48]. Future research and policy efforts should seek to develop evidence-based guidelines for AI-assisted screening that are consistent across diverse practice settings.

Current AI systems exhibit technical and practical limitations that may limit screening implementation. The AI system is unable to compare with prior radiographs [18], which is one of the critical features of screening programs. It also draws attention to the poor generalizability of this model to other populations of patients and the effect of demographic variables on AI performance [48]. Our findings demonstrate that successful implementation will require an understanding of both technical and human factors, such as variability in the dependence of performance on the different AI user interfaces [43]. On the other hand, radiologists tend to reject the correct AI detections occasionally [45]. Thus, robust validation studies, user-centered design, and performance monitoring are needed to realize the full potential of AI in lung cancer screening.

The role in lung cancer screening programs looks promising concerning AI but with careful optimization. The high negative predictive value (98.9%) for normal vs abnormal chest radiography [14] may make this a useful triaging tool in high-volume screening settings. Despite this, it is clear that the risk of missed diagnosis, in particular for nodule types and locations [18,33], dictates that AI should supplement, but not replace, the role of the radiologist. Future development in this area should be targeted toward better detection of the subtle findings, better generalizability between populations, and seamless integration into clinical workflow while maintaining the balance between sensitivity and specificity, which is needed for a successful lung cancer screening program. The transformative potential of AI in the early detection and management of lung cancer will depend on ongoing research, multi-stakeholder collaboration, and evidence-based policy initiatives.

There are several key limitations to this systematic review. The included studies were predominantly retrospective cross-sectional and thus may introduce selection bias and do not include the impact of AI on real-time clinical decision-making. Longitudinal follow-up studies are missing to rate patient outcomes in early cancer detection and mortality reduction. Most of the previous studies adopt the binary classification approach (detection or non-detection) on clinical diagnosis, which may oversimplify this complexity. In addition, there are only limited studies on the effect of AI on radiologist training, a lack of standardized reporting on performance-influencing factors, and few studies on practical clinical implementation difficulties, all of which limit the generalizability and clinical translation of findings.

## 5. Conclusions

This review shows the great promise of AI algorithms to improve lung nodule detection on chest radiographs. AI systems have better sensitivity than radiologists, especially for smaller or subtle nodules, and can be an excellent instrument in lung cancer screening programs. The impact of how those tools can be deployed in parallel with radiologists and produce a combined effect is what makes AI for radiology important in enhancing diagnostic accuracy and efficiency. Despite this, many obstacles in AI implementation remain, including inconsistency in AI performance across studies and characteristics of nodules studied, variability in evaluation methods used, and a balance between high sensitivity and an acceptable false-positive rate.

More effort should be directed toward future studies constructing screening-oriented AI algorithms that yield more consistent and generalizable results. More long-term comparative investigations of the effectiveness of AI-assisted screening for the patients’ outcomes and cost-effectiveness analyses are needed. AI deployment in clinical settings must enhance the value that both human expertise and AI can bring to clinical practices as technology advances. Data privacy and algorithmic bias, among other ethical considerations, should also be effectively addressed. The proposed strategies aim to enhance early detection rates and patients’ well-being regarding lung cancer diagnosis by using AI appropriately in radiology practice.

## Figures and Tables

**Table 1:** Research questions and motivations for evaluating AI performance in CXRs versus radiologists in lung nodule detection.

Research Questions	Motivation
How does the overall performance of AI algorithms compare to that of radiologists in detecting lung nodules in CXRs?	To assess the current state of AI technology in medical imaging and determine if AI can match or surpass human performance in this critical diagnostic task.
How does the performance of AI systems and radiologists vary across different nodule sizes, morphology types, and locations of lung nodules?	To identify the strengths and limitations of AI systems and radiologists across different nodule characteristics in diverse clinical scenarios.
What factors contribute to discrepancies between AI and radiologist interpretations?	To pinpoint areas where AI and human expertise differ, which can lead to insights for improving both AI algorithms and radiologist training.
What are the key patterns of missed lung nodule findings when comparing AI algorithms to radiologists in CXR interpretation across varying levels of radiologist experience and different types of nodules?	To identify specific areas where either AI or radiologists tend to make errors, considering factors such as nodule characteristics and radiologist experience.

Notes: Abbreviation: AI, Artificial Intelligence; CXRs, chest X-rays; Research questions were formulated using the PICO framework; Motivation was aligned with current clinical needs in radiology practice.

**Table 2:** Publication trends in AI-assisted lung nodule detection research.

Year	Number of Research	References
2020	6	[13,15,30,32,33,38]
2021	6	[35,44,49,50]
2022	5	[14,25,31,36,37,40]
2023	13	[16,17,23,24,26,27,28,29,34,39,43,45,48]
2024	4	[18,42,46,47]

Notes: This table shows an overview of the distribution of 34 included studies published over 2020–2024. References from the same year indicate different studies published in the same calendar year.

## Data Availability

The data in this systematic review are primary data obtained from scholarly articles that are cited in this review paper and published within the studies. The extraction and analysis protocols are reported in detail in the Materials and Methods section of the study. Specific additional data or analysis protocols may be requested from the corresponding author.

## Abbreviations

The following abbreviations are used in this manuscript:

AI	Artificial Intelligence
AUROC	Area under the receiver operating characteristic
AUC	Area under the curve
CAD	Computer-aided detection
CXR	Chest X-ray
DLAD	Deep learning-based automatic detection
GGO	Ground-glass opacity
LDCT	Low-dose computed tomography
NPV	Negative predictive value
PPV	Positive predictive value
